# Bradycardia in a newborn with accidental severe hypothermia: treat or don’t touch? A case report

**DOI:** 10.1186/s13049-021-00909-y

**Published:** 2021-07-12

**Authors:** Astrid Kuonen, Thomas Riva, Gabor Erdoes

**Affiliations:** Department of Anaesthesiology and Pain Medicine, Inselspital, Bern University Hospital, University of Bern, Freiburgstrass, CH-3010 Bern, Switzerland

**Keywords:** Accidental hypothermia, Neonate, External rewarming, Hyperglycemia

## Abstract

**Background:**

Hypothermia significantly affects mortality and morbidity of newborns. Literature about severe accidental hypothermia in neonates is limited. We report a case of a neonate suffering from severe accidental hypothermia. An understanding of the physiology of neonatal thermoregulation and hypothermia is important to decide on treatment.

**Case presentation:**

A low-birth-weight newborn was found with severe accidental hypothermia (rectal temperature 25.7 °C) due to prolonged exposure to low ambient temperature. The newborn presented bradycardic, bradypnoeic, lethargic, pale and cold. Bradycardia, bradypnea and impaired consciousness were interpreted in the context of the measured body temperature. Therefore, no reanimation or intubation was initiated. The newborn was closely monitored and successfully treated only with active and passive rewarming.

**Conclusion:**

Clinical parameters such as heart frequency, blood pressure, respiration and consciousness must be interpreted in light of the measured body temperature. Medical treatment should be adapted to the clinical presentation. External rewarming can be a safe and effective measure in neonatal patients.

## Background

Neonatal hypothermia is associated with significant mortality and morbidity [1]. Available literature about accidental hypothermia in newborns is limited [2]. We present the case of a newborn with severe accidental hypothermia (rectal temperature of 25.7 °C) due to prolonged exposure to low ambient temperature. In our case report we discuss the physiological hypothermia-related findings and the treatment of hypothermia, hyperglycemia, bradycardia and bradypnea in this newborn.

## Case presentation

### Case

A neonate weighing 2140 g, with a gestational age between 36 and 38 weeks, was found in a cardboard box, nude, covered only with a blanket. The ambient air temperature was recorded as 1 °C. A passer-by alerted the national emergency hotline. Fifteen minutes later an ambulance team with two paramedics arrived. The neonate was lethargic, but reacted to pain and opened its eyes. The paramedics carried the newborn to the ambulance. The blanket was carefully removed and rewarming was started by wrapping the infant in a warm towel. A pulse oximeter was installed and a rescue helicopter with an emergency physician was dispatched to the site. Fifteen minutes later the Helicopter Emergency Medical Services crew arrived.

**Fig. 1:**
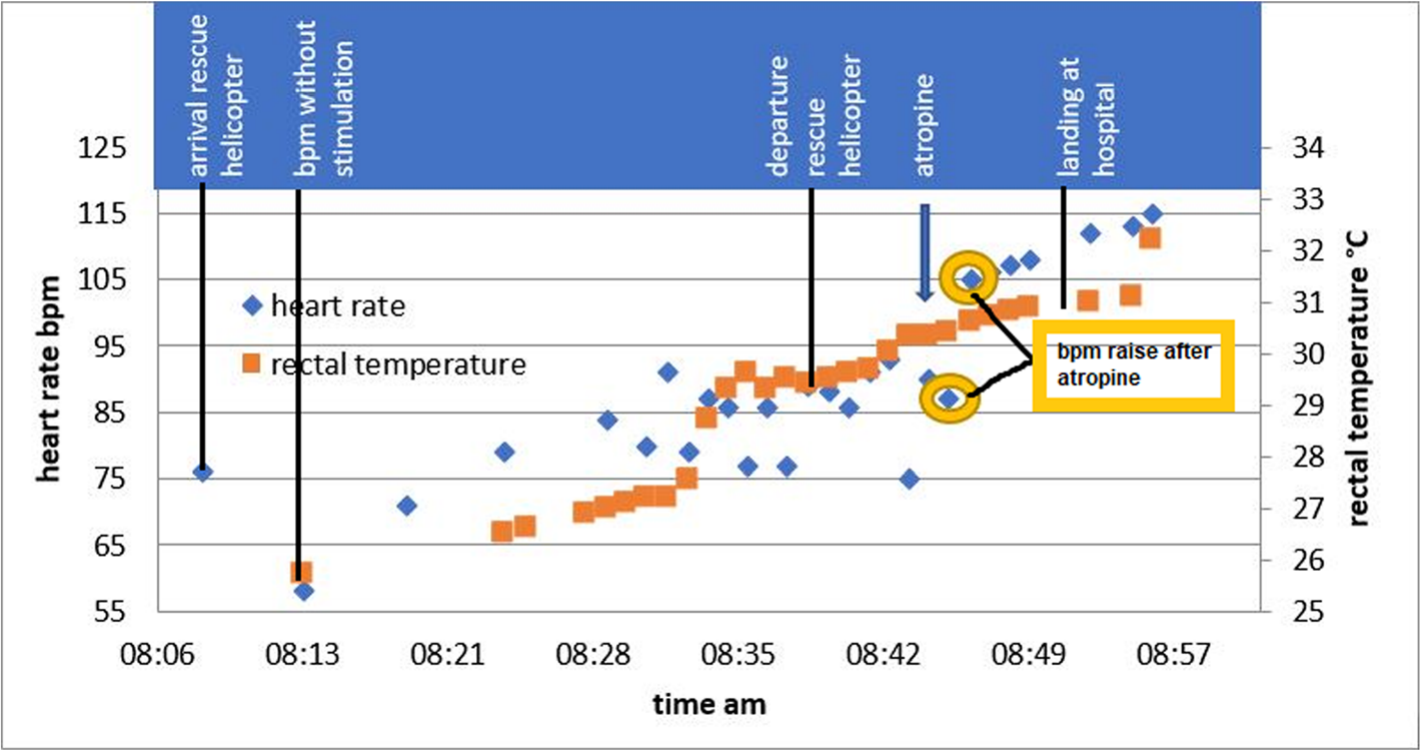
The course of rectal temperature and heart rate during transportation to the hospital

The newborn was breathing spontaneously without any signs of respiratory distress at a rate of 26 breaths per minute. Peripheral oxygen saturation was 97 %. The neonate was bradycardic, with a sinus rhythm of 76 beats per minute (bpm). Non-invasive blood pressure was 72/42 mmHg. The infant’s pupils were dilated. Repeated measurement of rectal temperature revealed an average of 25.7 °C (Fig. 1). No other injuries were detected.

Stress-induced transient hyperglycemia of 18.6 mmol l^− 1^ was noted. Peripheral i.v. access was not obtained on the first attempt, but tibial intra-osseous (i.o.) access (Arrow® EZ-IO R PD 15 mm) was successful. The neonate received a bolus of 20 ml kg^− 1^ of heated crystalloids mixed with glucose. Its core temperature rose 1.2 °C after the bolus (Fig. 1, Time 08:34). To avoid rescue collapse or arrhythmia, the newborn was carefully transported in a horizontal position to a tertiary referral center. To avoid any further heat loss, the air in the helicopter was heated, and the body was wrapped in warm blankets, with an electrical heating blanket on top. Heating pads surrounded by compresses were placed on the groin, the torso, and the head. The patient’s rewarming was surprisingly quick (7 °C in 1 h). Rectal temperature and heart rate during transport to the hospital are shown in Fig. 1. The neonate remained relatively bradycardic, but no arrhythmia was observed, and respiration remained sufficient during this time. The infant’s heart rate was unstable, falling intermittently under 80 bpm during rewarming (Fig. 1). Atropine sulfate 0.1 mg i.v. was administered in an attempt to increase the heart rate when the body temperature reached 30 °C. The heart rate rose from 87 bpm to 105 bpm (Fig. 1).

On arrival in the emergency department, the patient’s core temperature had risen to 32.2 °C. All vital signs were stable (blood pressure 90/47 mmHg, heart rate 115 min^− 1^, peripheral oxygen saturation 97 %). The initial capillary blood gas measured at 37 °C showed a pH of 7.25, base excess of 7.4 mmol L^− 1^, lactate of 5.2 mmol l^− 1^, and blood glucose of 19.8 mmol l^− 1^ (357 mg dl^− 1^). During further rewarming in the pediatric intensive care unit the neonate continued to breathe spontaneously and remained hemodynamically stable. Normal body temperature (36.5 − 37.5 °C) was reached within 6 h. Twelve hours after the initial resuscitation the neonate began to develop respiratory distress syndrome, which was successfully treated with high-flow nasal cannula therapy. The infant was transferred to the neonatology ward and was able to leave the hospital without any complications after seven days. We received permission from the legal guardian to publish this case for medical teaching.

### Literature review

The World Health Organization (WHO) defines neonatal hypothermia as mild at a core temperature of 36.0–36.5 °C, moderate at 32–36 °C and severe below 32 °C [3]. In neonates, hypothermia is associated with significant mortality and with morbidity such as sepsis, metabolic acidosis, respiratory distress syndrome, hypoglycemia and intra-ventricular hemorrhage [1, 4]. Neonates are prone to rapid heat loss because they have a large ratio of surface area to volume, decreased subcutaneous fat mass, greater body water content, and immature, thin skin, leading to increased evaporative water and heat losses [1, 5]. When a newborn is exposed to a cold environment, it attempts to maintain its body temperature by increasing cellular metabolism through voluntary muscle activity, and chemical, non-shivering thermogenesis caused by brown fat tissue and vasoconstriction [6].

Increased cellular metabolism in neonates leads to increased glucose utilization, rapid depletion of glycogen stores, and in particular a risk of hypoglycemia. The body counteracts hypoglycemia by decreasing insulin production while simultaneously increasing glucagon, epinephrine, growth hormone, and cortisol secretion. This may contribute initially to hyperglycemia, as seen in our case. During rewarming, there is increased responsiveness to insulin and increased glucose consumption, leading to a higher risk of hypoglycemia [5–7]. Therefore, dextrose-containing fluids were given to this hyperglycemic newborn. Close monitoring of the blood glucose level is required during rewarming. Our patient’s blood glucose level dropped in the pediatric intensive care unit from 17.8 mmol/l to 5.3 mmol/l within 2 h without medical intervention. The increased cellular metabolism also leads to increased oxygen consumption and pulmonary vasoconstriction. Accompanied by hyperventilation, the hypothermia-induced leftward shift of the hemoglobin-binding curve is increased, preventing adequate oxygen release from hemoglobin and exacerbating tissue-level hypoxia. In combination with catecholamine-induced peripheral vasoconstriction, anaerobic metabolism may increase lactate and metabolic acidosis [6].

Ensuring that the airway is patent and that ventilation and oxygenation are effective is central to the management of the critically ill or injured child [8]. The newborn in our case was bradypnoeic, but did not show any signs of respiratory distress, cyanosis, or recurrent apnea. Furthermore, hypoxia was not detected. Therefore, we observed the clinical development and frequently re-assessed it, with advanced airway management tools on standby. In the out-of-hospital setting, bag-mask ventilation rather than tracheal intubation or insertion of a supraglottic airway may be recommended [4, 8, 9]. Indications for intubation or laryngeal mask are respiratory failure or ineffective bag-mask ventilation [4, 8]. The use and timing of tracheal intubation will depend on the skill and experience of the available resuscitators. If the skills or equipment needed for safe intubation are lacking, the laryngeal mask is a valid alternative airway device [4]. Even for the pediatric anesthesiologist, tracheal intubation in neonates and small infants is a technically difficult skill that must be mastered with the highest possible first-attempt success rate and the largest possible margin of safety. Neonates are prone to hypoxemia, which occurs within seconds after cessation of spontaneous or assisted ventilation [10]. The younger the child, the shorter the duration of apnea before desaturation occurs [11]. In emergent situations, the complication rate associated with intubation increases substantially [12–14], resulting in treatment with the potential to do more harm than good.

The heart rate is reduced by around 10 bpm/°C reduction in core body temperature through a decrease in spontaneous depolarization of the pacemaker cells [6]. A heart rate of 70–80 bpm is therefore physiological at a core temperature of 25 °C in a neonate. As a result, in view of maintained perfusion, no CPR was initiated. In resuscitation guidelines it is stated that no drugs should be given until the patient has been warmed to a core temperature ≥ 30 °C due to the slowed drug metabolism, which can lead to potentially toxic plasma concentrations of any drug given [15]. The cardiac output in a neonate depends primarily on the heart rate. As our newborn was too bradycardic at a core temperature of 30° C and the heart rate was falling intermittently under 80 bpm, atropine 0.1 mg i.o. was given tentatively. Under this treatment, the increase in heart rate was adequate and no further reduction was detected (Fig. 1).

In accidental hypothermia, many arrhythmias (e.g., bradycardia, atrioventricular blocks, atrial fibrillation, nodal rhythms, and QRS prolongation with or without Osborn J waves) are considered benign. These usually disappear with patient rewarming and do not require further treatment, provided the perfusion is deemed adequate [7]. However, although atropine was effective in our case, we wouldn’t recommend giving it to all hypothermic newborns. According to the current guidelines, atropine can be used for bradycardia caused by increased vagal tone or anticholinergic drug toxicity. Epinephrine may be administered to infants and children when bradycardia and poor perfusion are unresponsive to ventilation and oxygenation [8, 9]. In the case of poor or absent perfusion, CPR should be started.

Our neonate’s rewarming rate was quick (7 °C in 1 h). The core temperature had already increased 1.2 °C after a bolus of 20ml/kg heated crystalloids (Fig. 1). There is no recommendation as to whether slow (less than 0.5 °C/h) or rapid (0.5 °C/h or more) rewarming is preferable [1]. The rate of rewarming did not affect the critical and important outcomes [4]. Neonatal resuscitation guidelines issued by Perlman et al. in [1] recommended distinguishing between the warming of infants with iatrogenic hypothermia at birth (which is generally of short duration) and infants in whom therapeutic hypothermia has been intentionally induced over 72 h. Slow rewarming is recommended in the case of therapeutic hypothermia (0.5 °C/h) [1]. In reported cases, the external rewarming rate in accidental hypothermia ranged from 0.5 °C/h to 10 °C/h [7]. During rewarming, the electro-cardiogram and core temperature should always be monitored [15].

Regardless of core temperature, vital sign abnormalities and changes in level of consciousness are the most important factors to consider when treating accidental hypothermia [7]. Successful out-of-hospital resuscitation requires rapid attention to supportive care (airway, breathing, circulation), clinical assessment, and treatment of injuries or other medical conditions. These are dependent on effective rewarming with active external rewarming techniques (forced air rewarming, radiant heat, chemical heat pads) and minimally invasive rewarming techniques (warmed crystalloids) [7]. Out-of-hospital management of severe hypothermia includes minimal and cautious movements in a horizontal position to avoid life-threatening arrhythmias or rescue collapse [7].

## Conclusions

Recommendations for neonatal life support exist, but they do not cover treatment and rewarming of newborns with severe accidental hypothermia in an out-of-hospital situation. There are differences between accidental hypothermia and hypothermia induced therapeutically, as well as between in-hospital and out-of-hospital settings. Emergency physicians and anesthesiologists, who may only infrequently be called on to care for such a patient, should be aware that clinical parameters such as heart rate, blood pressure, respiration and consciousness must be interpreted in the light of the core body temperature measured. Although some hypothermic neonates may require more aggressive resuscitation, a combination of external and internal rewarming can be an effective measure to treat severe accidental hypothermia. Medical treatment should be carried out as cautiously as possible to avoid arrhythmia or rescue collapse, and providers should pay close attention to airway management in order to help prevent critical events.

An understanding of the physiology of neonatal thermoregulation and hypothermia is needed to decide whether treat or don’t touch is the better approach in a newborn with severe accidental hypothermia.

## Data Availability

The datasets are available from the corresponding author on reasonable request.

## Abbreviations

°C: Degrees Celsius
g: Gram
mmHg: Millimeters of mercury
bpm: Beats per minute
%: Percent
mmol l-1: Millimoles per liter
i.v.: Intravenous
i.o.: Intraosseous
ml kg-1: Milliliters per kilogram
min-1: Per minute
mg: Milligram
pH: Potential of hydrogen ions
mg dl-1: Milligrams per deciliter
WHO: World Health Organization
bpm/°C: Beats per minute per degree Celsius
CPR: Cardiopulmonary resuscitation
≥: Greater than or equal to sign
Fig.: Figure
°C/h: Degrees Celsius per hour
